# TRK-fused gene (TFG) regulates ULK1 stability via TRAF3-mediated ubiquitination and protects macrophages from LPS-induced pyroptosis

**DOI:** 10.1038/s41419-022-04539-9

**Published:** 2022-01-28

**Authors:** Jian-Hong Shi, Chen Ling, Ting-Ting Wang, Li-Nuo Zhang, Wen-Wen Liu, Yan Qin, Ying-Hui Tan, Nai-Peng Cui, Zhi-Yu Ni

**Affiliations:** 1 Central Laboratory, Hebei Key Laboratory of Cancer Radiotherapy and Chemotherapy, Affiliated Hospital of Hebei University, Baoding, Hebei China; 2 Research Unit of Digestive Tract Microecosystem Pharmacology and Toxicology, Chinese Academy of Medical Sciences, Beijing, China; 3 Department of Breast Surgery, Affiliated Hospital of Hebei University, Baoding, Hebei China; 4 Obstetrics and Gynecology Department, Affiliated Hospital of Hebei University, Baoding, Hebei China; 5 Hebei University, Baoding, Hebei China

**Keywords:** Ubiquitylation, Inflammatory diseases

## Abstract

TRK-fused gene (TFG) is known to be involved in protein secretion and plays essential roles in an antiviral innate immune response. However, its function in LPS-induced inflammation and pyroptotic cell death is still unknown. Here, we reported that TFG promotes the stabilization of Unc-51 like autophagy activating kinase (ULK1) and participates in LPS plus nigericin (Ng) induced pyroptotic cell death. Our results showed that TFG-deficient THP-1 macrophages exhibit higher mitochondrial ROS production. LPS/Ng stimulation triggers a much higher level of ROS and induces pyroptotic cell death. ULK1 undergoes a rapid turnover in TFG-deficient THP-1 cells. TFG forms complex with an E3 ligase, tumor necrosis factor receptor-associated factor 3 (TRAF3), and stabilizes ULK1 via disturbing ULK1-TRAF3 interaction. Knockdown of TFG facilitates the interaction of ULK1 with TRAF3 and subsequent K48-linked ULK1 ubiquitination and proteasome degradation. Rescue of ULK1 expression blocks LPS/Ng-induced cell death in TFG-deficient THP-1 macrophages. Taken together, TFG plays an essential role in LPS/Ng-induced pyroptotic cell death via regulating K48-linked ULK1 ubiquitination in macrophages.

## Introduction

Inflammatory responses and cell death are major hosts respond to infection. Macrophages mediate crucial innate immune responses via Toll-like receptors (TLRs) responding to extracellular stimuli and Nod-like receptors (NLRs) responding to cytosolic perturbations [1]. TLRs triggers downstream immune signaling, including nuclear factor κB (NF-κB) and mitogen-activated protein kinase (MAPK) signaling cascades and inducing expression of precursor forms of cytokines IL-1β and IL-18 [2]. NLRs stimulate caspase-1 activation and trigger a form of cell death called pyroptosis, which is accompanied by IL-1β processing and releasing [3, 4].

Disordered inflammatory response and fulminant pyroptotic cell death of macrophages are involved in many diseases, such as acute lung injury [5], arthritis [6, 7], SARS-CoV-2-associated cytokine storm [8, 9]. Blocking pyroptotic cell death signaling cascades may benefit patients with infectious and autoinflammatory diseases by limiting tissue damage. However, strategies to modulate pyroptotic cell death of macrophages are still very limited. Our and other research groups have previously identified tumor necrosis factor receptor-associated factor 3 (TRAF3), a TRAF family member with E3 ligase activity, plays an essential role in NLRP3 inflammasome and TLR induced pyroptosis in macrophages [10–14].

Proteomic profiling of the TRAF3 interactome network has predicted TRK-fused gene (TFG) may serve as an important interaction candidate of TRAF3 [15], however, much less is known about the function of TFG in innate immune responses. TFG, also known as trafficking from the endoplasmic reticulum (ER) to Golgi regulator, play important roles in multiple biological processes, including protein secretion [16–18], protein stability [19], autophagy [20, 21], antiviral innate immune response [22, 23], neurologic disorder [24], and ongogenesis [25]. TFG was first identified to fused to the 3’ end of NTRK1 and generate the TRK-T3 fusion transcript in papillary thyroid carcinoma [26]. TFG is also involved in several different fusion genes, including TFG-ALK [27], TFG-GPR128 [28], TFG-TEC [29], TFG-RET [30], and TFG-FGFR1 [31]. TFG functions as a momentous regulator in ER-to Golgi transport and COPII-trafficking pathway [16–18, 25, 32, 33], which is known to contribute to protein secretion [34], ER stress [35], and autophagy [36]. Furthermore, increasing evidence demonstrates TFG participates in antiviral innate immunity via regulating NF-κB [23, 37] and mTORC1 [22] signaling pathways.

Although very recent evidence reported that TFG functions as an ER-to-Golgi resident protein to facilitate TRAF3 interaction with MAVS/TBK1 and engages in RIG-I-like receptor (RLR)-dependent type I interferon (IFN) antiviral responds [22], the role of TFG in inflammation and pyroptotic cell death is still largely unknown. Here, we identify TFG participates in LPS, a TLR4 agonist, induced pyroptosis in macrophages. TFG-deficient macrophages exhibit a higher level of mitochondrial ROS levels and pyroptotic cell death in THP-1 cells. TFG-deficiency promotes unc-51 like autophagy activating kinase 1 (ULK1) degradation. TFG-TRAF3 complex interferes TRAF3-ULK1 interaction and TRAF3-mediated K48-linked ULK1 ubiquitination and protects pyroptotic cell death in THP-1 macrophages. This implies TFG-TRAF3 interaction functions as a protection regulatory step in ULK1 stabilization and LPS-induced pyroptosis in macrophages.

## Results

### TFG-deficiency promotes mitochondrial ROS production and LPS/Ng-induced pyroptosis in THP-1 cells

**Fig. 1:**
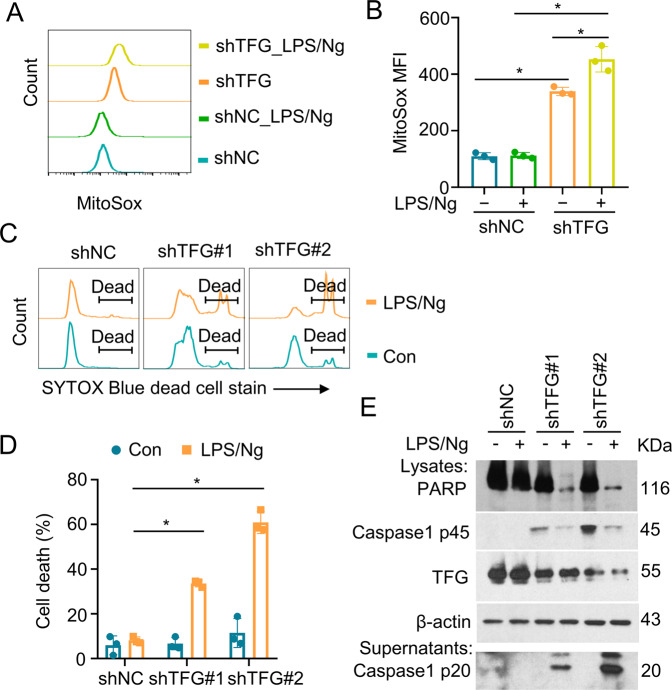
TFG deficiency promotes mitochondrial ROS production and LPS/Ng-induced pyroptosis in THP-1 cells. **A** THP-1 cells were transduced with a nontargeting control shRNA (shNC) or TFG-specific shRNAs (shTFG#1 and shTFG#2) to obtain TFG stable knockdown cells. Cells were primed with 1 μg/mL of LPS for 4 h followed by Nigericin (5 μg/mL) stimulation for an additional 60 min (LPS/Ng). SYTOX Blue Dead Cell staining and flow cytometry analysis were performed to analyze cell death. **B** Scatter dot plots of cell death percentages in groups described in **A** are shown. Bars represent the mean ± s.d. *p < 0.05; *n* = 3 for each group. **C** THP-1 cells were transduced with shNC or shTFG#2 were primed with LPS (1 μg/mL) for 4 h followed by Nigericin (5 μg/mL) stimulation for an additional 10 min. Cells were incubated with MitoSox for 15 min before harvest. Cells were then subjected to cytometry analysis to detect Mitochondria-associated ROS levels. **D** Mitochondrial ROS levels were presented as median fluorenscent intensity (MFI) and scatter dot plots of MitoSox MFI in the four groups described in **C** are shown. Bars represent the mean ± s.d. *p < 0.05; *n* = 3 for each group. **E** Control or TFG-knockdown THP-1 cells were treated with LPS (1 μg/mL) for 4 h followed by Nigericin (5 μg/mL) stimulation for 60 min. Whole-cell lysates and supernatants were subjected to IB. Experiments were independently repeated three times.

TFG is known to play a vital role in secretory cargo traffic from the ER to the Golgi apparatus [17, 18] and participate NF-κB signaling [37], which are essential processes in macrophage-mediated inflammation. To define the role of TFG in macrophage inflammatory function, we generated TFG stable knockdown THP-1 cells. TFG-deficiency significantly increased mitochondrial ROS production in THP-1 cells (Fig. 1A, B, shNC vs. shTFG). Elevated mitochondrial ROS is reported to be linked to pyroptosis [38]. We next primed THP-1 cells with LPS (1 μg/mL) for 4 h followed by Nigericin (5 μg/mL) stimulation to induce inflammasome and pyroptosis. LPS/Ng treatment triggered a higher level of mitochondrial ROS (Fig. 1A, B, shTFG vs. shTFG LPS/Ng) and promoted pyroptotic cell death (Fig. 1C, D) in TFG-knockdown THP-1 cells. Immunoblotting revealed that pro-caspase-1 p45 and cleaved caspase-1 p20 in the supernatant dramatically increased in TFG-knockdown cells (Fig. 1E). Moreover, PRAR degradation was observed in TFG-knockdown THP-1 cells after LPS/Ng stimulation (Fig. 1E).

Next, we analyzed the expression pattern of TFG and pyroptosis-associated genes caspase-1 and NLRP3 using GEO datasets (GSE10220), including 48 macrophage and 48 monocyte samples from 86 patients with symptoms of the acute coronary syndrome. As shown in Supplementary Fig. S1, TFG expression level was lower in monocytes than that in macrophages, whereas the expression levels of pyroptosis-associated genes, caspase-1 and NLRP3, were higher in monocytes than that in macrophages. These results indicated that TFG is negatively correlated with pyroptosis-associated genes, caspase-1, and NLRP3.

### TFG-deficiency downregulates autophagy-associated kinase ULK1 and increases p62 protein level

**Fig. 2:**
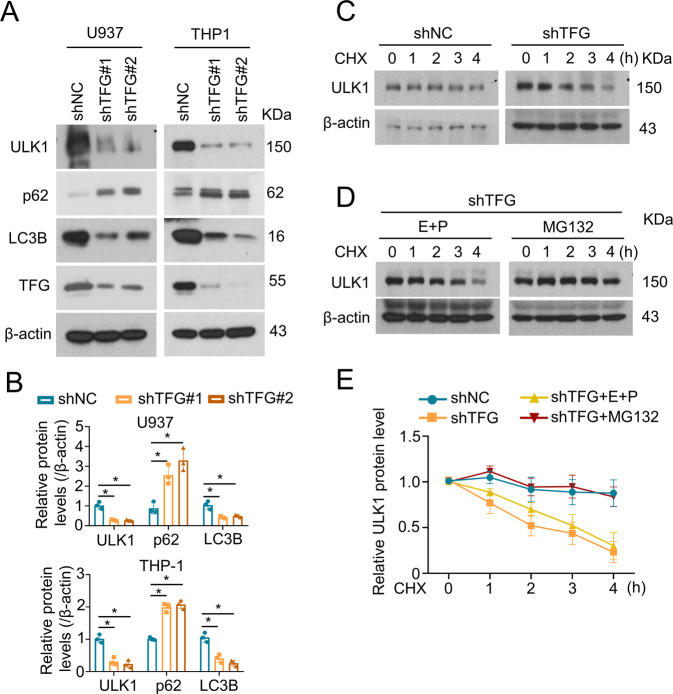
TFG deficiency downregulates autophagy-associated kinase ULK1 and increases p62 protein level. **A** THP-1 and U937 cells were transduced with shNC, shTFG#1, or shTFG#2. IB were performed to analyze ULK1 and p62 expression levels. **B** Relative ULK1 and p62 protein levels are shown as bar plots. Bars represent the mean ± s.d. *p < 0.05; *n* = 3 for each group. **C** THP-1 cells transduced with shNC or shTFG#2 were treated with CHX (20 μg/mL) for indicated times and IB assays were performed to analyze ULK1 degradation. **D** TFG-knockdown THP-1 cells (shTFG#2 transduced) were pretreated with lysosome inhibitor E64D (10 μg/mL) + pepstatin A (1 μg/mL) (E + P) or proteasome inhibitor MG132 (10 μg/mL) for 2 h. Cells were then treated with CHX (20 μg/mL) for indicated times and IB assay were performed to analyze ULK1 degradation. **E** Relative protein levels of ULK1 are shown. Data are shown as mean ± s.d.; *n* = 3 for each group. Experiments were independently repeated three times.

Our group [10] and others [39] have previously identified ULK1 negative regulates mitochondrial ROS and pyroptotic cell death. We next analyzed the role of TFG in autophagy-associated proteins, ULK1, p62, and LC3B. TFG deficiency dramatically reduced ULK1 and LC3B protein levels and increased p62 levels in THP-1 and U937 cells (Fig. 2A, B). ULK1 undergoes a rapid turnover in macrophages [10]. To analyze ULK1 stabilization in TFG-knockdown macrophages, control and TFG-deficient THP-1 cells were treated with protein synthesis inhibitor CHX for 0–4 h and ULK1 protein levels were analyzed using immunoblotting. TFG deficiency resulted in rapid degradation of ULK1 in THP-1 cells (Fig. 2C and E). To further clarify the mechanism of TFG-mediated ULK1 degradation, THP-1 cells were pretreated with lysosome inhibitor E64D + pepstatin A or proteasome inhibitor MG132 followed by CHX treatment. The result demonstrated that blocking the proteasome system by MG132, not lysosome inhibitor, significantly inhibited ULK1 degradation in TFG-deficient THP-1 cells (Fig. 2D, E).

We further analyzed TFG and ULK1 expression correlation using online database GEPIA Correlation Analysis tools (http://gepia.cancer-pku.cn/detail.php?clicktag=correlation). As shown in Supplementary Fig. S2, in whole blood samples from GTEx datasets and in human tumor tissue samples (thymoma and lune adenocarcinoma) from TCGA datasets, TFG and ULK1 pair-wise gene correlation analysis confirmed that TFG expression positive correlated with ULK1 (whole blood: R = 0.73, *P* = 0; thymoma tissue: R = 0.62, *P* = 4.7e-14; lung adenocarcinoma tissue: R = 0.47, *P* = 0).

### TFG-deficiency facilitates ULK1 ubiquitination and proteasome degradation

**Fig. 3:**
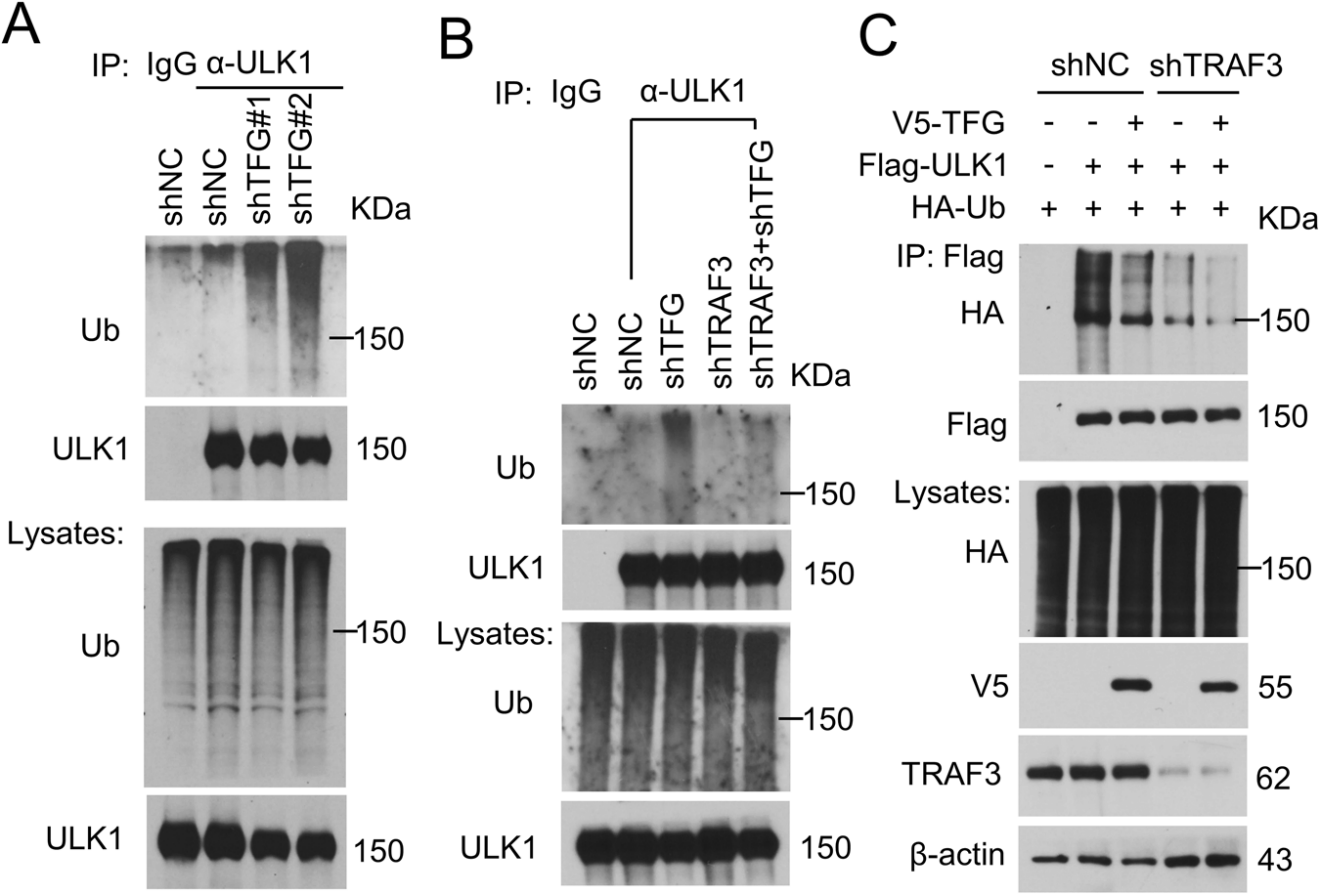
TFG deficiency facilitates ULK1 ubiquitination and proteasome degradation. **A** THP-1 cells were transduced by shNC, shTFG#1, and shTFG#2 and treated with MG132 for 2 h before harvest. ULK1 was isolated by IP and subjected to IB assay using anti-ubiquitin (Ub) and anti-ULK1 antibodies. Whole-cell lysates were also subjected to direct IB (bottom panels). **B** THP-1 cells transduced with shNC, shTFG, shTRAF3, and shTFG+shTRAF3 were treated with MG132 for 2 h before harvest. ULK1 was isolated by IP and subjected to IB assays using anti-Ub and anti-ULK1 antibodies. **C** V5-TFG, Flag-ULK1, and HA-Ub were transfected into shNC- or shTRAF3-transduced HEK293 cells as indicated. Flag-ULK1 were isolated by IP and subjected to IB assays as indicated. Protein lysates were also subjected to direct IB (bottom panels). Experiments were independently repeated three times.

To further reveal the mechanism of ULK1 degradation, the ubiquitination of ULK1 in control or TFG-knockdown THP-1 cells was investigated. THP-1 cells were pretreated with MG132, a proteasome inhibitor, to block ubiquitin-mediated degradation and the ubiquitination of ULK1 was detected. TFG-deficiency significantly increased ULK1 ubiquitination in THP-1 cells (Fig. 3A). Our group previously identified E3 ligase TRAF3 mediates K48-linked ubiquitination and protein degradation of ULK1 in macrophages [10]. We next generated TFG-TRAF3 double knockdown THP-1 macrophages and detected whether TRAF3 participates in TFG-associated ULK1 ubiquitination regulation. Knockdown of TRAF3 disturbed ULK1 ubiquitination in TFG-deficient THP-1 cells (Fig. 3B, lane 5 vs. lane 3), which indicated that TRAF3 participates in TFG-mediated ULK1 ubiquitination in macrophages. To further confirm the role of TRAF3 in TFG-mediated ULK1 ubiquitination, control or TRAF3-deficient HEK293 cells were transfected with TFG, ULK1, and ubiquitin expression vector, and ULK1 ubiquitination analysis was performed. TFG overexpression reduced ubiquitinated ULK1 levels in shNC-transduced HEK293 cells (Fig. 3C, lane 3 vs. lane 2). The ubiquitinated ULK1 level significantly decreased in shTRAF3-transduced cells (Fig. 3C, lane 4 vs. lane 2), and TFG expression couldn’t further abate ULK1 ubiquitination (Fig. 3C, lane 5 vs. lane 4). These results suggested that TFG exists as an inhibitor in the TRAF3-mediated ULK1 ubiquitination process.

### PB1 domain of TFG and TRAF domain of TRAF3 mediates the association of TRAF3 and TFG

**Fig. 4:**
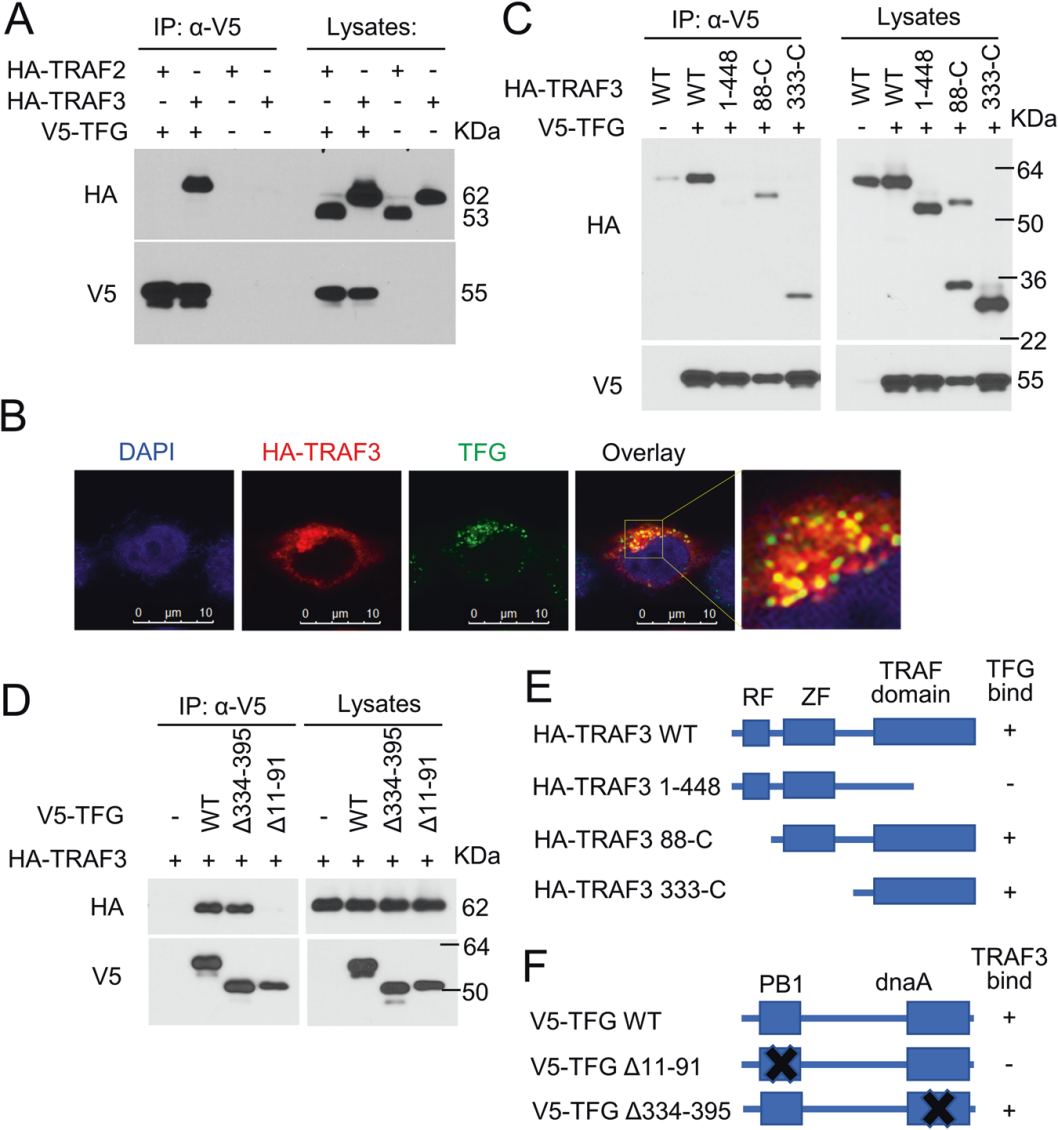
PB1 domain of TFG and TRAF domain of TRAF3 mediates association of TRAF3 and TFG. **A** HEK293 cells were co-transfected with HA-TRAF2, HA-TRAF3, and V5-TFG. Co-IP assays were performed. TRAF2, another TRAF family member, served as a negative control. **B** HEK293 cells were transfected with HA-TRAF3. Colocalization of overexpressed HA-TRAF3 (red) and endogenous TFG (green) were analyzed by confocal microscopy. Nuclear was visualized by DAPI staining (blue). **C** V5-tagged TFG was transfected into HEK293 cells together with the indicated TRAF3 mutants. The physical interaction of TGF with the different TRAF3 mutants was determined by co-IP assays (left panel). The expression of TRAF3 mutants and TFG was monitored by direct IB using anti-HA and anti-V5 antibodies (right panel). **D** HA-tagged TRAF3 was transfected into HEK293 cells together with the indicated TFG mutants. The physical interaction of TFG with the different TRAF3 mutants was determined by co-IP assays. **E, F** Schematic diagram of wild-type (WT) form and deletion mutants of TRAF3 (**E**) and TFG (**F**). Their TFG (**E**) and TRAF3 (**F**) binding ability (+) or deficiency (−) is summarized based on the results presented in **C** and **D**. RF RING finger, ZF TRAF-type zinc finger, PB1 PB1_TFG domain, dnaA dnaA domain. Experiments were independently repeated three times.

We next examined the association of TFG and TRAF3. CoIP assay demonstrated that TFG interacted with TRAF3 in HEK293 cells (Fig. 4A). Confocal microscopy assay demonstrated that TFG colocalized with TRAF3 in the cytosol of HEK293 cells (Fig. 4B). To map the domain of TRAF3-TFG physical interaction in mammalian cells, TFG and TRAF3 mutant vectors were co-transfected into HEK293 cells (Fig. 4C-F). Wild-type (Fig. 4C, lane 2) and TRAF3 lacking RF domain (88-C, Fig. 4C, lane 4) and lacking ZF domain (333-C, Fig. 4C, lane 5) had the TFG binding ability. The TRAF3 mutant lacking TRAF domain (1–448, Fig. 4C, lane 3) was unable to bind to TFG, indicating the TRAF domain of TRAF3 is necessary for TFG-TRAF3 interaction. Meanwhile, wild-type TFG (Fig. 4D, lane 2) and TFG mutant lacking dnaA domain (Δ334–395, Fig. 4D, lane 3) were capable of binding to TRAF3. TFG mutant lacking PB1 domain (Δ11–91, Fig. 4D, lane 4) was unable to bind to TRAF3, suggesting the important role of PB1 domain in TFG-TRAF3 interaction.

### TFG suppresses K48-linked ULK1 ubiquitination and proteasome degradation

**Fig. 5:**
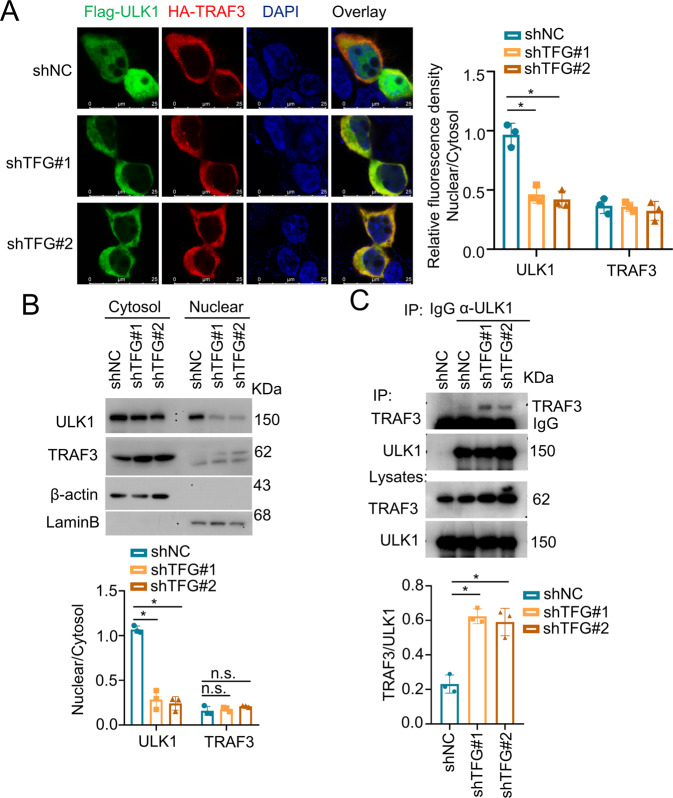
TFG regulates ULK1 subcellular localization. **A** HEK293 cells were transduced with a nontargeting control shRNA (shNC) or TFG-specific shRNAs. Cells were transfected with Flag-ULK1 and HA-TRAF3. Colocalization of overexpressed Flag-ULK1 (green) and HA-TRAF3 (red) were analyzed by confocal microscopy. Nuclear was visualized by DAPI staining (blue). Right panel shows relative fluorescence density of nuclear/cytosol ratio of ULK1 and TRAF3. Data are shown as mean ± s.d. *p < 0.05; *n* = 3 for each group. **B** THP-1 cells were transduced with shNC or TFG-specific shRNAs. Nuclear and cytosol fractions from THP-1 cells were extracted and subjected to IB using anti-ULK1 and anti-TRAF3 antibodies. Lower panel shows nuclear/cytosol ratio of ULK1 and TRAF3. Data are shown as mean ± s.d. *p < 0.05; n.s. no significance. **C** Co-IP assay of endogenous ULK1 and TRAF3 in control or TFG-specific knockdown THP-1 cells. Lower panel shows TRAF3/ULK1 ratio in which indicates binding activity. Data are shown as mean ± s.d. *p < 0.05; *n* = 3 for each group. Experiments were independently repeated three times.

To investigate the role of TFG and TRAF3 in the regulation of ULK1 ubiquitination, subcellular localization and confocal microscopy analysis were performed. Flag-ULK1 expression vector was transfected together with HA-TRAF3 into control or TFG-deficient HEK293 cells and colocalization were analyzed using confocal microscopy. In the control cells (shNC), ULK1 staining was distributed throughout the cytosol and nucleus (Fig. 5A, upper panel). Whereas in the TFG-deficient cells, we observed that ULK1 accumulated in the cytosol and colocalized with TRAF3 (Fig. 5A, lower panel). Then, cytosol and nuclear extracts were prepared and immunoblotting was performed to examine the effect of TFG on ULK1 subcellular distribution. Nuclear localization of ULK1 significantly decreased in TFG-deficient THP-1 cells (Fig. 5B). Moreover, the physical interaction activity of endogenous TRAF3 and ULK1 was dramatically downregulated after TFG was knockdown in THP-1 cells (Fig. 5C). Taken together, these results demonstrated that TFG regulates ULK1 nuclear/cytosol translocation and disturbs the interaction of TRAF3 and ULK1.

**Fig. 6:**
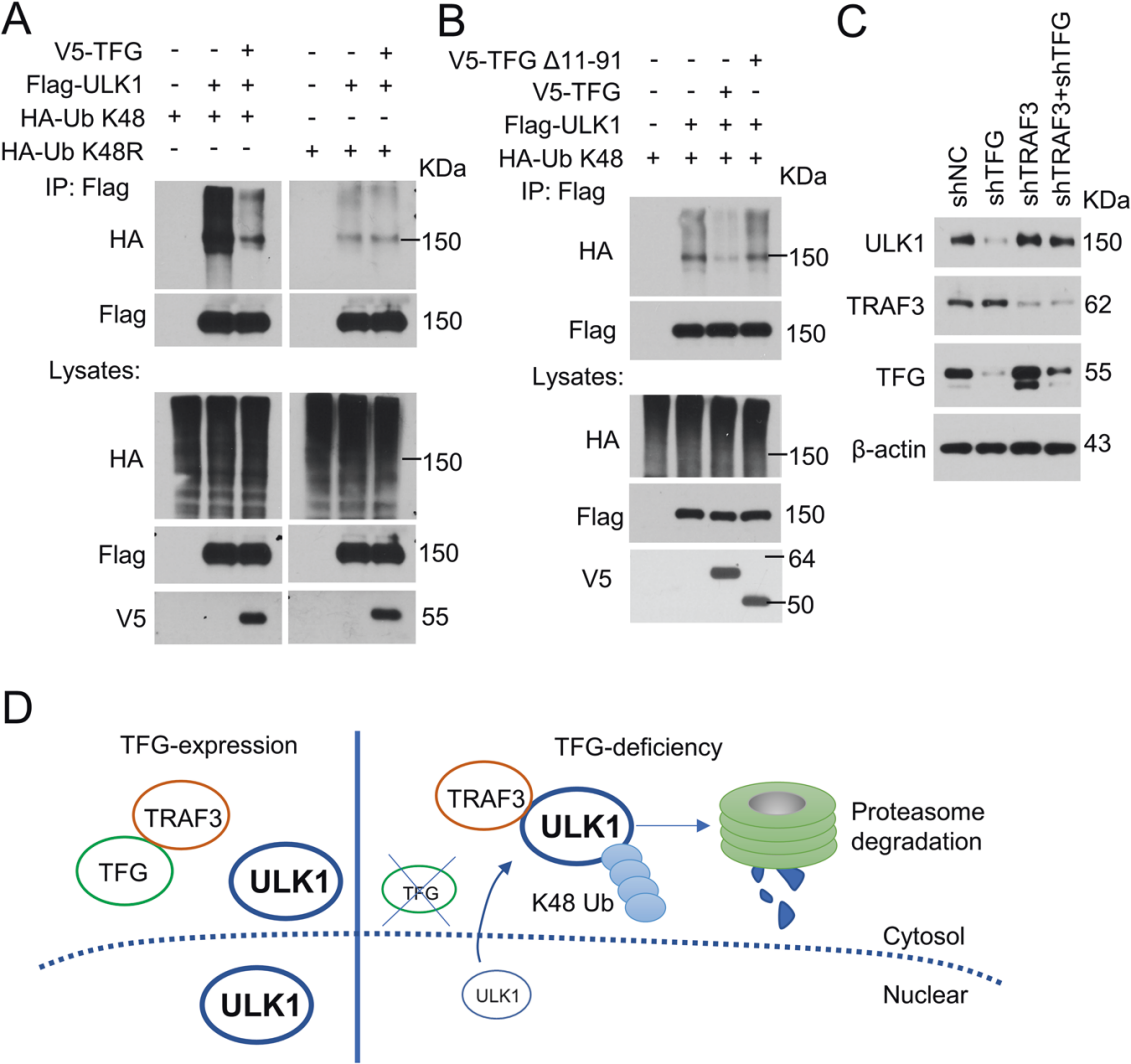
TFG suppresses TRAF3-mediated ULK1 degradation. **A** Flag-ULK1 and V5-TFG were transfected into HEK293 cells together with HA-Ub-K48 or HA-Ub K48R as indicated. Flag-ULK1 was isolated by IP and subjected to IB assays using anti-HA and anti-Flag antibodies. Protein lysates were also subjected to direct IB (bottom panels). **B** Flag-ULK1 and HA-Ub K48 were transfected into HEK293 cells together with V5-tagged wild-type TFG or mutant TFG (Δ11–91, lacking TRAF3 binding domain). Flag-ULK1 were isolated by IP and subjected to IB using anti-HA and anti-Flag antibodies. Protein lysates were also subjected to direct IB (bottom panels). **C** THP-1 cells were transduced by shNC, shTFG, and/or shTRAF3. Total cell lysates were extracted and ULK1 protein level were analyzed by IB assay. **D** A proposed model of TFG regulates TRAF3-mediated ULK1 ubiquitination and proteasome degradation. Experiments were independently repeated three times.

Next, we analyzed the role of TFG in ULK1 proteasome degradation. Overexpression of V5-tagged TFG in HEK293 cells significantly reduced K48-linked ULK1 polyubiquitination level (Fig. 6A, left panels), but not single-lysine mutant K48R-linked ubiquitination (Fig. 6A, right panels). Expression of V5-TFG Δ11–91 (TFG mutant lacking TRAF3 interaction domain) failed to decrease ULK1 ubiquitination (Fig. 6B). We next examined ULK1 expression in THP-1 cells transduced by shNC, shTFG, and/or shTRAF3. TFG-deficiency decreased ULK1 protein level (Fig. 6C, lane 2 vs. lane 1). However, the knockdown of TFG in TRAF3-deficient THP-1 cells could not change ULK1 protein level (Fig. 6C, lane 4 vs. lane 3). These results indicated that TFG deficiency facilitates TRAF3-mediated K48-linked ULK1 ubiquitination and proteasome degradation.

### Rescue of ULK1 expression blocks LPS/Ng-induced cell death in TFG-deficient THP-1 cells

**Fig. 7:**
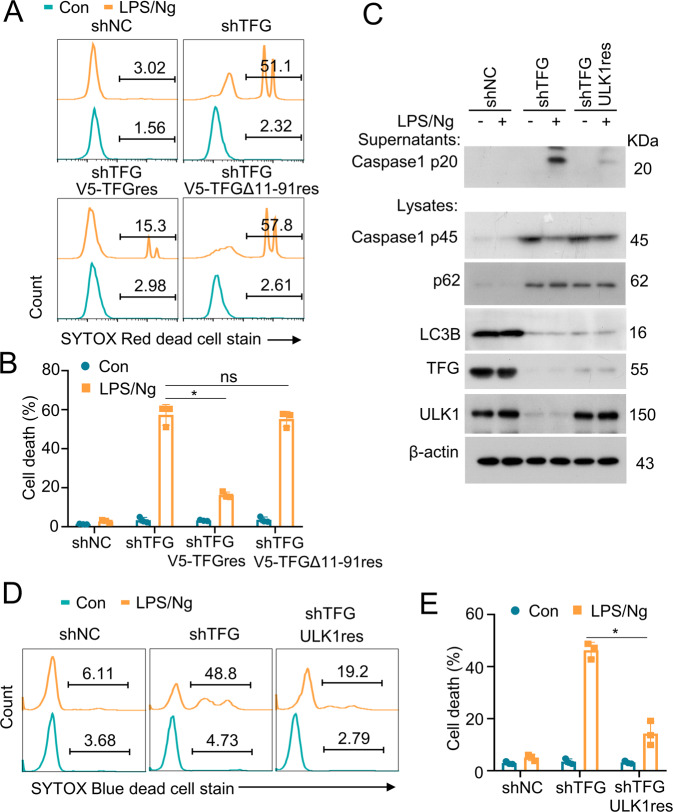
Rescue of ULK1 expression blocks LPS/Ng-induced cell death in TFG-deficient THP-1 cells. **A** THP-1 cells were transduced with shNC or shTFG. TFG stable knockdown THP-1 cells were then transfected with V5-TFG rescue or V5-TFG Δ11–91 rescue expression vectors to rescue TFG (shTFG + V5-TFGres) or TFG Δ11–91 expression (shTFG + V5-TFGΔ11-91res). Cells were stimulated with LPS/Ng and analyzed by SYTOX Red dead cell staining. **B** Scatter dot plots of cell death percentage in groups described in **A** are shown. Data are shown as mean ± s.d. *p < 0.05; n.s. no significance; *n* = 3 for each group. **C** THP-1 cells were transduced with shNC or shTFG. TFG stable knockdown THP-1 cells were then transfected with LV8N-(mcherry)-ULK1 to rescue ULK1 expression (shTFG + ULK1res). Cells were then treated with LPS/Ng and whole-cell lysates and supernatants were subjected to IB. **D** THP-1 cells were stimulated with LPS/Ng. Cell death were analyzed by SYTOX Red dead cell staining. **E** Scatter dot plots of cell death percentage in groups described in **D** are shown. Data are shown as mean ± s.d. *p < 0.05; *n* = 3 for each group. Experiments were independently repeated three times.

To explore the role of TFG in LPS/Ng-induced pyroptosis, TFG lentiviral rescue expression vector and TFG Δ11–91 mutant rescue expression vector were transduced into TFG stable knockdown cells to rescue TFG expression (Supplementary Fig. S3). Cells were then stimulated with LPS/Ng and cell death analysis revealed that rescue of wild-type TFG significantly reduced LPS/Ng-induced cell death, while the rescue of a mutant form of TFG Δ11–91, lacking TRAF3 binding domain, couldn’t alleviate LPS/Ng-induced pyroptosis (Fig. 7A, B).

To further reveal the role of ULK1 in TFG-mediated cell death, ULK1 lentiviral expression vectors were transduced into TFG stable knockdown THP-1 cells to rescue ULK1 protein expression. Cells were treated with LPS/Ng and cleaved caspase-1 p20 levels in the supernatant were significantly reduced in shTFG + ULKres cells (Fig. 7C). Moreover, cell death percentage was significantly decreased in shTFG + ULK1res group compared with that in shTFG group (Fig. 7D, E). Although we found that autophagy-associated protein LC3B decreased and p62 increased in TFG-knockdown THP-1 and U937 cells (Fig. 2A) and the further study demonstrated that Earle’s Balanced Salt Solution (EBSS) starvation significantly increased LC3B-II levels in shNC-transduced THP-1 cells (Supplementary Fig. S4), LPS/Ng treatment didn’t induce LC3B-II production or p62 degradation (Fig. 7C). These results suggested that ULK1 played a vital role in LPS/Ng-induced cell death in an autophagy-independent manner in THP-1 cells.

**Fig. 8:**
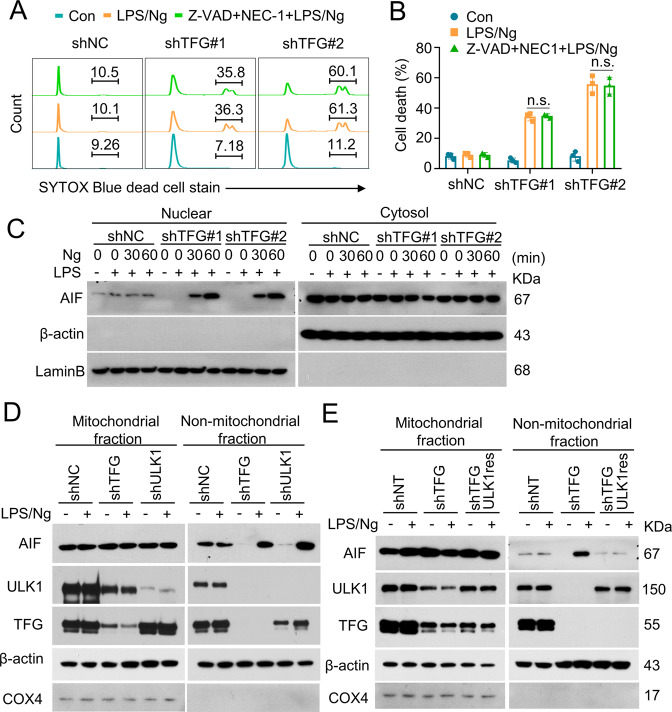
AIF participates in LPS/Ng-induced cell death. **A** THP-1 cells (shNC and shTFGs) were stimulated with LPS (1 μg/mL) for 4 h followed by Nigericin (5 μg/mL) stimulation for 60 min (LPS/Ng). Z-VAD plus NEC-1 were used to block apoptosis or necrosis. Cell death were determined using SYTOX staining. **B** Scatter dot plots of cell death percentage in groups described in **A** are shown. Data are shown as mean ± s.d. n.s. no significance; *n* = 3 for each group. **C** THP-1 cells were transduced with TFG-specific shRNAs. Cells were stimulated with LPS/Ng. Nuclear/Cytosol fractions were extracted and IB assays were performed to analyze AIF localization. **D** THP-1 cells were transduced with shNC, shTFG, or shULK1. Cells were stimulated with LPS/Ng. Mitochondrial/non-mitochondrial fractions were extracted and IB assays were performed to analyze AIF localization. COX4 was detected as a mitochondrial marker. **E** THP-1 cells were transduced with shNC or shTFG. TFG stable knockdown THP-1 cells were then transfected with LV8N-(mcherry)-ULK1 to rescue ULK1 expression (shTFG + ULK1res). Mitochondrial/non-mitochondrial fractions were extracted and IB assays were performed to analyze AIF localization. COX4 was detected as a mitochondrial marker. Experiments were independently repeated three times.

To analyze the mechanisms of TFG-associated cell death, THP-1 cells were pretreated with Z-VAD (a pan-Caspase inhibitor) and NEC-1 (a necroptosis inhibitor) before LPS/Ng stimulation. However, Z-VAD and NEC-1 pretreatment could not reduce LPS/Ng-induced cell death in TFG-deficient cells (Fig. 8A, B), which indicated that LPS/Ng-induced cell death in TFG-deficient THP-1 cells may not associate with apoptosis and necrosis process.

Mitochondrial apoptosis-inducing factor (AIF) translocation from mitochondrial to nuclear is known to play a vital role in caspase-independent cell death [40–42]. We next examined AIF subcellular distribution. Knockdown of TFG promoted AIF nuclear translocation in LPS/Ng-treated THP-1 cells (Fig. 8C). Moreover, we also observed increased AIF protein levels in non-mitochondrial fraction after LPS/Ng stimulation in both TFG-knockdown- and ULK1-knockdown-THP-1 cells (Fig. 8D), which suggested AIF releasing from mitochondrial upon LPS/Ng-induced cell death. Moreover, rescue ULK1 protein expression blocked LPS/Ng-induced AIF releasing from mitochondrial (Fig. 8E). Taken together, these results demonstrated that mitochondrial-nuclear trafficking of AIF plays a role in LPS/Ng-induced cell death in TFG-deficient cells.

## Discussion

Pattern recognition receptors (PRRs) identify repetitive motifs of pathogens such as LPS of gram-negative bacteria and trigger innate immune responses [43]. With the activation of TLRs and NLRs, macrophages trigger crucial programmed pyroptotic cell death via caspase-1-dependent processing and release abundant pro-inflammatory cytokines such as IL-1β [44]. The balance between the positive and negative regulation of pyroptotic cell death and inflammatory factor releasing is required to ensure the most favorable outcome for the host. Post-translational modifications (PTMs), including phosphorylation and polyubiquitination, of inflammatory signaling molecules, have been shown to control innate immunity and pyroptotic cell death of innate immune cells [43, 45]. In this study, we revealed the role of TFG in the pyroptosis process via regulating ULK1 polyubiquitination modification and stabilization.

TFG is known to participate in NF-κB signaling pathway [37, 46], which contributes to oxidative stress and inflammatory responses. Our present data demonstrated that TFG-deficient THP-1 macrophages exhibit a higher level of mitochondrial ROS production and LPS/Ng-induced caspase-1 activation and pyroptotic cell death. Moreover, ULK1 protein appears a rapid degradation in TFG-deficient THP-1 and U937 macrophages. Recent evidence reported that ULK1, a central regulator on autophagy [47], plays an essential role in NLRP3 inflammasome activation and pyroptosis process in macrophages [10], hepatocytes [39], and microglial cells [48]. Using ULK1 rescue cell model, we revealed the regulation function of TFG in caspase-1 processing and subsequent pyroptosis due to its crucial role in mediating ULK1 degradation in macrophages.

Ubiquitin ligase-mediated PTMs play a critical role in regulating the autophagy pathway, thereby contributing to cell death. ULK1 is a key regulator of autophagy and mitophagy processes and it undergoes a rapid turnover in multiple cell contexts [49–51]. Both K48- [52–54] and K63-linked [55–57] ubiquitination modifications have been reported to regulate ULK1 stabilization and activation. However, the role of TFG on ULK1 degradation is largely unknown. Our previous research demonstrated that TRAF3, an E3 ubiquitin ligase, promotes K48-linked ULK1 ubiquitination and degradation [10]. Very recent evidence reported a novel interaction of TFG with TRAF3, which allows the efficient recruitment of TRAF3 to its substrate TANK-binding kinase (TBK1) [22]. In the present data, we demonstrated TFG recruits TRAF3 in HEK293 cells and THP-1 macrophages. The existence of TFG interrupts the binding of TRAF3 with its substance ULK1 and thereby inhibits TRAF3-mediated K48-linked polyubiquitination and proteasome degradation of ULK1.

AIF is considered to conduct DNA degradation in a range of cell death stimuli [58]. AIF is anchored to the inner mitochondrial membrane. AIF releases from the mitochondrial membrane into the cytosol and is subsequently imported to the nucleus to participate cell death process [59]. In the current results, we observed AIF releases from mitochondrial and translocates into nuclear in response to LPS plus Ng stimulation in TFG-deficient THP-1 cells. Rescue of ULK1 expression blocks AIF translocation and LPS/Ng-induced cell death in TFG-deficient cells.

In summary, this study proposes an important role of TFG in ULK1 subcellular localization and TRAF3-ULK1 interaction, which is required for K48-linked ULK1 ubiquitination and ULK1/AIF axis mediated pyroptosis.

## Methods

### Antibodies and reagents

Antibodies for PARP (9532, 1:1000), ULK1 (8054, 1:1000), p62 (5114, 1:1000), AIF (5318, 1:1000) and Lamin B (12586, 1:1000) were purchased from Cell Signaling Technology (MA, USA). TFG (ab156866, 1:10,000) and GSDMD (ab210070, 1:1000) were from Abcam. Caspase-1 (AG-20B-0048-C100, 1:1000) were purchased from AdipoGen (CA, USA). HA (12CA5, 1:1000 for IP) and HA-HRP (3F10, 1:5000) were from Roche Life Science (Switzerland). Flag (F3165, 1:500 for IP) and Flag-HRP (A8592, 1:5000), LC3B (L7543, 1:1000) and β-actin (A2228, 1:10,000) were from Sigma–Aldrich (MO, USA). Ub (P4D1, 1:1000), TRAF3 (H-122, 1:1000), Tubulin (SC-8035, 1:1000), and rabbit IgG (sc-2027, 1:1000) were from Santa Cruz Biotechnology (CA, USA). COX4 (ARG66326, 1:1000) were purchased from Arigo Biolaboratories. V5 (R96025, 1:500 for IP) and V5-HRP (R96125, 1:5000) were from Thermo Fisher Scientific (MA, USA).

MitoSox™ Red Mitochondrial Superoxide Indicator (M36008), SYTOX™ Blue Dead Cell Stain (S34857), and SYTOX™ Red Dead Cell Stain (S34859) were purchased from Thermo Fisher Scientific (MA, USA). LPS, Nigericin, Z-VAD-FMK (Z-VAD), Z-Leu-Leu-Leu-al (MG132) were purchased from Sigma–Aldrich (MO, USA). Necrostatin-1 (NEC-1) was purchased from ApexBio (Texas, USA). Cycloheximide (CHX), E-64d, and Pepstatin A were purchased from Cayman Chemical (Michigan, USA).

### Cell culture

Human embryo kidney 293 (HEK293) cells, human monocyte THP-1, and U937 cells were obtained from Cell Resource Center (IBMS, CAMS/PUMC, Beijing, China). HEK293 cells were cultured in Dulbecco’s modified Eagle’s medium (DMEM) containing 10% of FBS (Gemini Bio, USA), 100 U/mL of penicillin, and 100 μg/mL of streptomycin. Human monocyte THP-1 and U937 cells were cultured in endotoxin-free RPMI 1640 medium containing 10% FBS, 100 U/mL of penicillin, and 100 μg/mL of streptomycin. All cells were incubated at 37 °C in a humidified atmosphere of 5% CO_2_. For THP-1 and U937 cells, before stimulation, cells were induced differentiation to macrophages using 30 ng/mL PMA in RPMI 1640 with 0.5% FBS for 24 h.

### Gene expression constructs and shRNAs

Expression plasmids encoding HA-TRAF2, HA-TRAF3 (wild-type and truncation mutants 88-C, 333-C and 1–448), Flag-ULK1, HA-Ub (wild-type and mutants K48 and K48R) were from Addgene (Watertown, MA, USA). V5-TFG expression plasmid was from DNASU Plasmid (Tempe, AZ, USA). Truncation mutants of V5-TFG (Δ11–91 and Δ334–395) were constructed using QuikChange multi-site-directed mutagenesis kit (Agilent Technologies, La Jolla, CA, USA) according to the manufacturer’s instructions. Primers used to generate V5-TFG Δ11–91 were 5’-gatctaagtgggaagggccagccaagaccc-3’ (sense) and 5’-gggtcttggctggcccttcccacttagatc-3’ (anti-sense), and primers used to generate V5-TFG Δ334–395 were 5’-gcacaaacttacactgcccaaactggacctggttatc-3’ (sense) and 5’-gataaccaggtccagtttgggcagtgtaagtttgtgc-3’ (anti-sense).

Lentiviral expression construct LV8N-(mcherry)-ULK1 was purchased from GenePharma (Suzhou, China). Lentiviral silencing vectors targeting human TFG (shTFG#1, shTFG#2), and human shULK1 were purchased from GE healthcare (CO, USA). Lentiviral silencing vectors targeting human TRAF3 (shTRAF3) were purchased from Sigma-Aldrich. The sequences of shRNAs are listed below: shTFG#1, TGCTGTTGACAGTGAGCGCCGAAATAAAGTGA ATCGTTTATAGTGAAGCCACAGATGTATAAACGATTCACTTTATTTCGATGCCTACTGCCTCGGA; shTFG#2, TGCTGTTGACAGTGAGCGCAACCAAGATGAAATCAATAA ATAGTGAAGCCACAGATGTATTTATTGATTTCATCTTGGTTTTGCCTACTGCCTCGG; shULK1, TGCTGTTGACAGTGAGCGCGCCCTTTGCGTTATATTGTATTAGTGAAGCCACAGATGTAATACAATATAACGCAAAGGGCATGCCTACTGCCTCGGA; shTRAF3, CCGGCCTTGGCCGTTTAAGCAGAAACTCGAGTTTCTGCTTAAACG GCCAAGGTTTTTG.

For V5-TFG rescue (V5-TFGres) expression vector construction, the targeting sequence of shTFG#2 was mutated synonymously from ‘CAA GAT GAA ATC AAT’ to ‘CAG GAC GAG ATA AAC’.

For lentiviral vector-mediated transduction, HEK293 cells were used for lentiviral particles preparation. In brief, lentiviral shRNA vectors encoding nontargeting control shRNA (shNC) or specific RNAs were transfected into HEK293 cells along with packaging plasmids psPAX2 and pMD2.G. Forty-eight hours after transfection, the supernatants containing packaged viruses were collected and used to transduce target cells. The transduced cells were selected using puromycin or sorted by flow cytometric cell sorting system based on lentiviral shRNA vector carried GFP or mcherry expression.

### Flow cytometry

For mitochondria-associated ROS analysis, cells were incubated with MitoSOX (M36008, Thermo Fisher Scientific, USA) at 5 μM for 15 min at 37 °C and washed with PBS solution and resuspended in cold PBS solution for Flow cytometry analysis using FACS Aria (BD Bioscience). For cell death assays, cells were harvested and resuspended in PBS containing 2% FBS and incubated with SYTOX™ Blue Dead Cell Stain (Thermo Fisher Scientific, USA) for 10 min at room temperature and then subjected to flow cytometry to quantify the dead cell population. Experiments were independently repeated three times.

### Subcellular colocalization by confocal microscopy

Cells were fixed with 4% (w/vol) paraformaldehyde and then permeabilized with 1% Triton-X100/PBS. The cells were blocked with 10% goat serum and incubated with indicated primary antibodies overnight at 4 °C. The slides were washed three times followed by incubation with FITC-or TRITC-conjugated secondary antibody. Slides were washed and mounted in antifade reagent with DAPI and pictures were taken with an SP5 RS confocal microscope (Leica, Wetzlar, Germany) and analyzed by SlideBook 5.0 software. Experiments were independently repeated three times.

### Cell lysates and subcellular extract preparation

For whole-cell lysates, 1 × 10^6^ cells were harvested and resuspended in 100 μL of RIPA buffer (Solarbio, Beijing, China) for 10 min at 4 °C and centrifuged at 8000 rpm for 10 min. The supernatants were obtained and subjected to IB analysis.

For mitochondrial fraction extraction, a Mitochondria/Cytosol Fractionation Kit (BioVision, CA, USA) was used and extracts were prepared according to the protocol. Briefly, 5 × 10^7^ THP-1 cells were collected and washed in cold PBS. Cells were then resuspended and lysed with Cytosol Extraction Buffer Mix containing DTT and protease inhibitors and homogenized in an ice-cold dounce tissue grinder on ice for 30–50 passes. The homogenate was centrifuged at 3000 rpm for 30 min at 4 °C and collected the supernatant to obtain cytosolic fraction. The pellets were then incubated in Mitochondrial Extraction Buffer Mix containing DTT and protease inhibitors, vortex for 10 seconds, and saved as a mitochondrial fraction.

For nuclear/cytosol fractions, THP-1 cells were harvest and lysed in cytosol lysis buffer (10 mM HEPES, pH 7.9, 10 mM KCl, 0.1 mM EDTA, 0.4% NP-40, 1 mM DTT, 0.5 mM PMSF) for 10 min on ice. Cells were spun down and the supernatant cytosol fraction was obtained. Then, the nuclear pellets were washed and lysed using nuclear extraction buffer (20 mM HEPES pH 7.9, 0.4 M NaCl, 1 mM EDTA, 1 mM DTT, 1 mM PMSF) for 15 min on ice to obtain the nuclear extract.

### Immunoblot (IB) and co-immunoprecipitation (co-IP)

Whole-cell lysates or subcellular extracts were prepared using RIPA buffer and the samples were separated by SDS-PAGE, transferred to PVDF membranes. The membranes were then blocked with 5% non-fat milk and incubated with a specific primary antibody and horseradish peroxidase-conjugated secondary antibody. Chemiluminescent detection (ECL, Roche Diagnostics, Penzberg, Germany) was used to visualize the immunoreactive bands followed by X-ray film exposure (Thermo Fisher Scientific, MA, US).

For co-IP assays, the cells were lysed in a buffer containing 20 mM HEPES (pH 7.6), 175 mM NaCl, 0.25% NP-40, 10% glycerol, 1 mM EDTA, 1 mM DTT, 1 mM PMSF, and protease inhibitor cocktail. Cell lysates were precleared with protein A-agarose (Santa Cruz Biotechnology, CA, USA). The supernatants were incubated with indicated antibodies for 1 h at 4 °C and then incubated with protein A-agarose for 2 h at 4 °C. The co-immunoprecipitated complex was washed and analyzed by IB. Each experiment was independently repeated three times.

### Ubiquitination assays

For endogenous protein ubiquitination assays, cells were pretreated with MG132, a proteasome inhibitor, for 2 h and then lysed with a NP-40 lysis buffer (50 mM Tris-HCl, pH 7.5, 120 mM NaCl, 1% NP-40, 1 mM EDTA, 1 mM DTT, 1 mM PMSF) containing 6 M urea and protease inhibitors. The indicated proteins were isolated by IP with indicated antibody and detected by IB using anti-ubiquitin antibody. For transfected ubiquitination, expression vectors along with wild-type or mutant HA-tagged Ubiquitin expression vectors (HA-Ub, HA-Ub-K48, HA-Ub-K48R) were transfected into HEK293 cells. The cells were pretreated with MG132 for 2 h and subjected to ubiquitination assays using anti-HA antibody. Experiments were independently repeated three times.

### Statistical analysis

Statistical analyses were performed using GraphPad Prism software version 8.0 (GraphPad Software, San Diego, CA, USA). Comparison of two groups was carried out using a two-tailed unpaired *t*-test, and comparison of more than two groups was carried out with one-way ANOVA and Tukey’s multiple comparisons. A *p*-value less than 0.05 is considered statistically significant.

## Supplementary information

Supplementary Figures S1-S4

aj-checklist

## Data Availability

The datasets generated during the current study are available from the corresponding author on reasonable request.
