# Supplementary material for: TRK-fused gene (TFG) regulates ULK1 stability via TRAF3-mediated ubiquitination and protects macrophages from LPS-induced pyroptosis
Source: Cell Death Dis. 2022 Jan 28;13(1):93. doi: 10.1038/s41419-022-04539-9 (PMC8795729; doi:10.1038/s41419-022-04539-9)
Supplement: Supplementary file 1 — Supplementary Figures S1-S4 [file 41419_2022_4539_MOESM1_ESM.pdf]

Figure S1

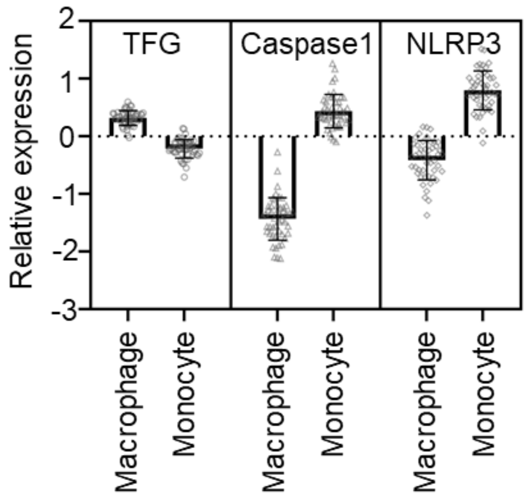

Supplementary Figure S1. TFG expression analysis in monocytes and macrophages. Expression pattern of TFG and pyroptosis-associated genes caspase1 and NLRP3 were analyzed using GEO datasets (GSE10220), including 48 macrophage and 48 monocyte samples from 86 patients with symptoms of acute coronary syndrome.

Figure S2

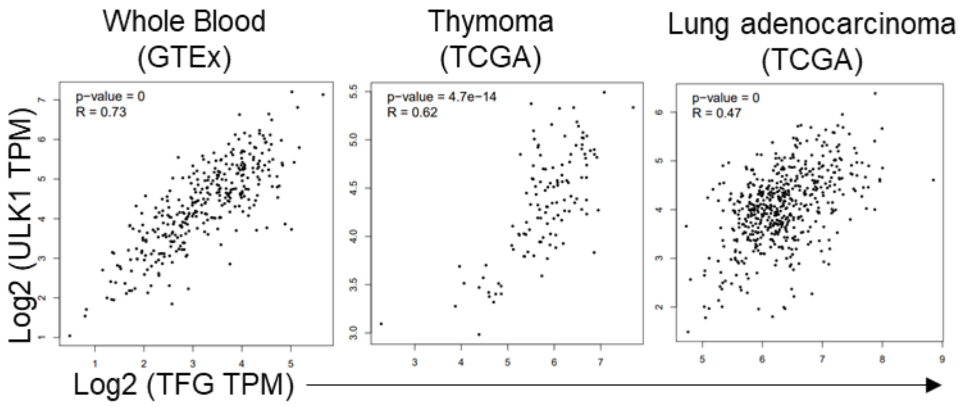

Supplementary Figure S2. TFG expression analysis in blood cells and tumor tissues. TFG and ULK1 pair-wise gene correlation in whole blood (Genotype-Tissue Expression, GTEx datasets), thymoma (The Cancer Genome Atlas, TCGA datasets) and lung adenocarcinoma (TCGA datasets) were analyzed using GEPIA Correlation Analysis tools (http://gepia.cancer-pku.cn/detail.php?clicktag=correlation).

Figure S3

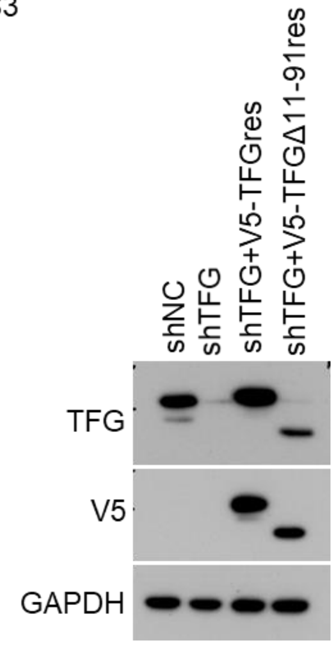

Supplementary Figure S3. TFG expression rescue in THP-1 cells. IB analysis of V5-TFG and V5-TFG Δ11-91 expression rescue in THP-1 cells transduced with a non-targeting control shRNA (shNC), TFG-specific shRNA#2 (shTFG), shTFG plus expression vectors, full length V5-TFG rescue (V5-TFGres) or V5-TFG Δ11-91 rescue (V5-TFG Δ11-91res).

Figure S4

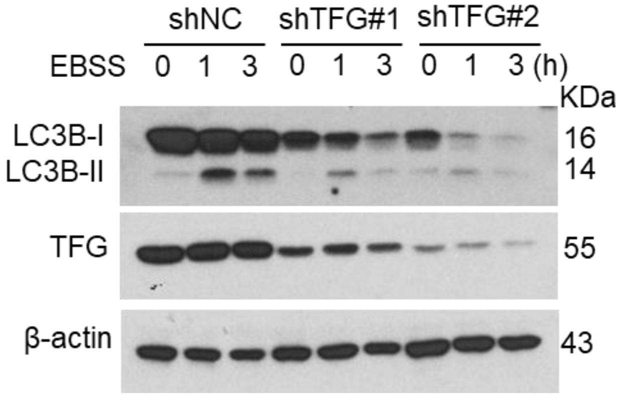

Supplementary Figure S4. EBSS starvation induced autophagy in control and TFG knockdown THP1 cells. THP-1 cells transduced with shNC, shTFG#1 or shTFG#2 were exposed to Earle's balanced salt solution (EBSS) media to induce autophagy by severe nutrient starvation for indicated times. Whole cell lysates were subjected to IB. Experiments were independently repeated three times.
